# Perceptions Toward a Smoking Cessation App Targeting LGBTQ+ Youth and Young Adults: A Qualitative Framework Analysis of Focus Groups

**DOI:** 10.2196/publichealth.6188

**Published:** 2016-11-18

**Authors:** N Bruce Baskerville, Darly Dash, Katy Wong, Alanna Shuh, Aneta Abramowicz

**Affiliations:** ^1^ Propel Centre for Population Health Impact Applied Health Sciences University of Waterloo Waterloo, ON Canada

**Keywords:** qualitative research, focus groups, mobile applications, telemedicine, sexuality, smoking cessation, smartphone

## Abstract

**Background:**

The prevalence of smoking among lesbian, gay, bisexual, trans, queer, and other sexual minority (LGBTQ+) youth and young adults (YYA) is significantly higher compared with that among non-LGBTQ+ persons. However, in the past, interventions were primarily group cessation classes that targeted LGBTQ+ persons of all ages. mHealth interventions offer an alternate and modern intervention platform for this subpopulation and may be of particular interest for young LGBTQ+ persons.

**Objective:**

This study explored LGBTQ+ YYA (the potential users’) perceptions of a culturally tailored mobile app for smoking cessation. Specifically, we sought to understand what LGBTQ+ YYA like and dislike about this potential cessation tool, along with how such interventions could be improved.

**Methods:**

We conducted 24 focus groups with 204 LGBTQ+ YYA (aged 16-29 years) in Toronto and Ottawa, Canada. Participants reflected on how an app might support LGBTQ+ persons with smoking cessation. Participants indicated their feelings, likes and dislikes, concerns, and additional ideas for culturally tailored smoking cessation apps. Framework analysis was used to code transcripts and identify the overarching themes.

**Results:**

Study findings suggested that LGBTQ+ YYA were eager about using culturally tailored mobile apps for smoking cessation. Accessibility, monitoring and tracking, connecting with community members, tailoring, connecting with social networks, and personalization were key reasons that were valued for a mobile app cessation program. However, concerns were raised about individual privacy and that not all individuals had access to a mobile phone, users might lose interest quickly, an app would need to be marketed effectively, and app users might cheat and lie about progress to themselves. Participants highlighted that the addition of distractions, rewards, notifications, and Web-based and print versions of the app would be extremely useful to mitigate some of their concerns.

**Conclusions:**

This study provided insight into the perspectives of LGBTQ+ YYA on a smoking cessation intervention delivered through a mobile app. The findings suggested a number of components of a mobile app that were valued and those that were concerning, as well as suggestions on how to make a mobile app cessation program successful. App development for this subpopulation should take into consideration the opinions of the intended users and involve them in the development and evaluation of mobile-based smoking cessation programs.

## Introduction

Significant reductions in tobacco prevalence and use have been achieved; however, tobacco use continues to be the leading cause of preventable disease, disability, and death in Canada. In 2014, 18.1% of Canadians aged 12 years and older were daily or occasional smokers [1] but this prevalence is not equal across all subpopulations. Smoking prevalence is particularly high in lesbian, gay, bisexual, trans, queer, and other sexual minority (LGBTQ+) persons with estimates ranging between 24% and 45% across different sexual orientation and gender identity groups [2]. There are even higher rates among LGBTQ+ youth and young adults (YYA) [2]. According to the 2013-2014 Canadian Community Health Survey (CCHS), 34.0% of homosexuals (interpret with caution due to marginal sampling variability) and 35.1% of bisexuals among 18- to 24-year-olds report smoking daily or occasionally compared with 23.3% of heterosexuals [3]. Further, 22% of high school-aged adolescents who identify as lesbian, gay, or bisexual report daily cigarette use compared with 11% of non-LGBTQ+ adolescents [4].

There are several suggested factors that contribute to high smoking rates among LGBTQ+ persons. These include minority stress, victimization, discrimination, harassment, abuse, mental health, targeted marketing by the tobacco industry, frequenting bars and nightclubs, other substance use, higher rates of stress, depression, and alcohol use, and low socioeconomic status [5-9].

Remafedi and Carol (2005) interviewed LGBTQ+ youth who highlighted the need for programs to be culturally specific and targeted toward youth, and that programming should involve LGBTQ+ in planning and implementation [10]. However, to date, there are few smoking cessation interventions and studies that specifically target LGBTQ+ YYA [11,12]. The majority of interventions are geared toward LGBTQ+ individuals of all ages and involve group cessation counseling or broader social marketing campaigns, where the interventions have been either tailored to LGBTQ+ people or assessed for effectiveness within the community. A recent review highlighted that group counseling programs showed evidence for effectiveness but had low reach in the target population [11]. Further, media and social marketing campaigns were found to be successfully implemented and increased media coverage and outreach to LGBTQ+ organizations; however, as they are primarily descriptive studies, there is limited evidence of impact [11].

Recently, digital technologies have shown promise [13,14] and offer potential avenues to deliver a smoking cessation program to LGBTQ+ YYA. For example, the mobile phone market is growing rapidly with statistics from the United States showing that mobile phone ownership in adults was 68% in 2015 [15]. In young persons (aged 18-29 years), ownership is as high as 86% and is closely reaching saturation [15]. Similar to the United States, Canadian data from 2015 indicate that, overall, 68% of adults own a mobile phone [16], while 2013 data indicate that 86% of 18- to 34-year-olds own a mobile phone [17]. Of interesting note, minorities and those with lower income levels are more likely to access the Internet primarily through mobile phones [18]. Given continual growing rates of mobile phone ownership across the population, mobile health (mHealth) interventions offer a potential method of engaging YYA who identify as LGBTQ+.

There are numerous benefits to delivering a smoking cessation program through a mobile phone app compared with traditional approaches. These include low cost of the intervention, accessibility, portability, personalization, self-monitoring capabilities, location-determining sensors, access to the Internet, and ability to connect with social networking platforms [19-21].

There is a growing body of evidence supporting mobile phone-based interventions for smoking cessation, although studies have primarily evaluated text messaging interventions for smoking cessation [13,20]. Research on mobile apps has been limited to small pilot studies [22-24] and trial protocols currently in progress [25-29]. Smoking cessation apps are also being developed and delivered rapidly as hundreds of apps are available on iPhone and Android platforms. Studies have found that publicly available cessation apps do not conform to established best practice guidelines including the 5A protocol: ask, advise, assess, assist, and arrange follow-up [30-32], do not recommend quitline use or have low rates for recommending approved medications [31-33], and do not take full advantage of interactive and proactive tailoring for the consumer [33]. However, there are no studies reporting whether LGBTQ+ YYA are interested in a mobile phone app for smoking cessation.

We addressed this gap in the literature by focusing on the LGBTQ+ YYA population. This study explored LGBTQ+ YYA (the potential users’) perceptions on a culturally tailored mobile app for smoking cessation. Potential users shared their opinions on this hypothetical cessation tool, along with opinions on what content and design features should be included.

## Methods

### Design and Recruitment

We conducted a total of 24 focus groups (FGs) among LGBTQ+ YYA in Toronto (n=18 groups) and Ottawa (n=6 groups) from March to May 2015. Recruitment was done using a variety of methods through purposive and snowball sampling: posting flyers and verbal announcements in spaces frequented by LGBTQ+ people; Facebook posts on LGBTQ+-friendly group pages; paid Facebook advertisements; emails from LGBTQ+ agencies; organizations making calls through social media channels; targeted physical recruitment at bars and nightclubs in Ottawa; and asking participants to refer eligible peers.

At the time of recruitment, interested participants contacted the project coordinator via email and completed an electronic demographic intake questionnaire. Physical questionnaires were available for those unable to complete the electronic form. Eligible participants were 16-29 years old, a part of the LGBTQ+ community, and a current smoker or recent quitter (defined as having not quit for more than 6 months before completing the intake questionnaire). Thus, those who had quit for more than 6 months or those who had never smoked were ineligible to participate. Those who were eligible were triaged into age, city, and LGBTQ+ groups for the purpose of organizing and hosting FGs based on location (ie, Toronto vs Ottawa), age (youth aged 16-18 years vs young adults aged 19-29 years), and gender or sexual identity of participants The objective was to create FG conditions that were the most conducive for open and safe dialogue among participants (eg, gay youths speaking with other gay youths). The project coordinator confirmed attendance and information about FGs via email and attended all sessions. The attendance rate at the FGs was 74% (204 individuals attended out of the 275 who agreed to attend).

### Focus Group Procedures

Facilitators and note takers (one each per location) were trained in study objectives and the semistructured FG script and protocol. The FG script with questions and description of the mHealth idea can be found in Multimedia Appendix 1. Facilitators and note takers identified as part of the community. Participants provided written consent. FGs were conducted in LGBTQ+ community safe spaces. Participants were provided with the mobile app intervention idea, as part of a broader program of research to explore potential interventions for LGBTQ+ YYA. The intervention idea was handed out on paper to each participant who were also provided with a pen and notepad to write down their thoughts about the LGBTQ+-specific mobile smoking cessation app intervention (Textbox 1). They were then asked to verbally describe and share their thoughts and feelings with the group. This included sharing perceived usefulness, relevance, likes and dislikes, concerns, and ideas or suggestions for further improvement of this intervention idea.

**Mobile app intervention idea for LGBTQ+ youth and young adults.**

Do you own a smartphone? Ever play Candy Crush or use Instagram? What if there was an app that could help you quit smoking designed specifically for LGBTQ+ youth and young adults? For example, this quit smoking app would allow you to create an individualized quit plan where you can set a quit date, it would provide feedback on how you’re doing, record what triggers you to smoke, and give you tips on how to remain smoke-free, as well as links to counselling services. One of the advantages of the app would be access to a peer support network which would connect you to other LGBTQ+ peers who are also trying to quit or who have already stopped smoking.

The app would be part of a bigger social media campaign that would include a webpage, Facebook page, YouTube videos, and Twitter feed with access to more detailed educational resources about smoking and quitting [eg, nicotine replacement therapy, like gum or the patch]. LGBTQ+ role models would promote the campaign.

The FGs contained 3-17 participants and lasted for 90 minutes. Participants were remunerated with a Can $50 cash incentive. All FGs were audio-recorded and professionally transcribed. This study was approved by a University of Waterloo Research Ethics Committee.

### Data Analysis

We qualitatively analyzed the data using a framework analysis technique that incorporated the stages of familiarization, identification of a thematic framework, indexing, charting, mapping, and interpretation [34]. Coding categories were developed and entered into the NVivo 11 (QSR International) software. To validate coding, 2 authors (AS and KW) independently coded 6 FG transcripts and then compared for consistency. Any discrepancies in coding were discussed and resolved with the principal investigator (NB). In this way, each author was able to critically challenge one another on differing perspectives and any potential biases.

After coding of the initial transcripts, a thematic framework was developed from the a priori issues and emerging codes by generating major themes and subthemes in relation to the original FG questions. Associated responses were categorized iteratively. To maintain the context of FG participant responses, they were listed under the questions from which they were derived and then categorized separately as a type of response. Throughout the coding or indexing process, regular meetings were held between 3 of the authors to discuss and refine the emerging thematic framework. Indexing was accomplished by coding each FG in NVivo 11, with reliability checked by a third author (DD) through review of the NVivo 11 file. At the final stage, the original responses were charted and grouped according to the finalized themes and subthemes. Member checking was completed with 14 participants to confirm findings. The final stage of mapping and interpretation involved comparing and contrasting the responses to search for patterns. Responses were grouped according to the final themes and subthemes that emerged from the data. Saturation of findings occurred after analysis of the fifteenth FG transcript. Representative quotes were selected from the FG responses to illustrate key themes and subthemes.

We used the Consolidated Criteria for Reporting Qualitative Research guideline statement to assist in the reporting of the study [35].

## Results

### Participants

A total of 204 participants were recruited in 24 FGs with 18 youth (aged 16-17 years) and 186 young adults (aged 19-29 years). We conducted 11 FGs with mixed gender and sexual orientation participants and 13 gender- or sexual orientation–specific groups (3 gay, 3 bisexual, 3 transgender, 2 lesbian, and 2 queer groups).

The majority of participants identified as white (48%, 115/241), female gender (39%, 85/218), bisexual orientation (27%, 57/209), and renting or owning their homes (50%, 118/234), and the majority completed high school (77%, 158/204). Also, 84% (171/204) of individuals were daily or occasional smokers, and 37% (75/204) of individuals reported smoking within 30 minutes after waking. Additional characteristics are depicted in Multimedia Appendix 2.

### Overview

We found that responses toward a culturally tailored smoking cessation app focused on 3 main areas: (1) why a mobile app for cessation would work; (2) concerns over the use of a mobile app; and (3) suggestions that should be incorporated into a mobile app for this population.

#### Reasons Why a Mobile App Would Work

The majority of participants in this study overwhelmingly expressed positive feelings toward a mobile smoking cessation app. Many supported this type of intervention and were excited about its potential.

I’m definitely most likely to use the app. That actually excites me thinking about it because I never really thought about that and people I know would use it – hands down, for sure.

FG02, Gay group participant

I think it would be an awesome idea for me if there were some apps for quitting smoking because I can’t leave my phone for one hour.

FG18, Mixed group participant

A number of specific factors made a smoking cessation app appealing for LGBTQ+ YYA. First, the most commonly expressed benefit was its potential for accessibility and availability at all times, without requiring a visit to see a counselor, physician, or other health professionals.

Yes, the (app) idea (is) going to work because I can access it at home as opposed to when I have to go out. I get access at any time, anywhere. I get it when I wake up first thing in the morning (and) before I go to bed.

FG06, Mixed group participant

Next, participants liked that apps could provide the mechanism to help them monitor and track their attempts and progress in changing behavior. Individuals reflected that this would allow them to understand their progress and notice patterns. In several instances, participants indicated that the app should be able to track the number of cigarettes consumed, the amount of money someone was saving while not smoking, and how long they had been smoke-free in hours and days.

I like this idea, I think it’s really cool. Having a way to track my smoking because often times I just don’t know how much (I smoke); I’m not paying attention to it. But if I had an app that I can check in on or sign into, I can probably keep track of it and eventually help me quit.

FG09, Lesbian group participant

An app that would help me is (one) that I could input on a daily basis how many cigarettes I’ve taken (per) day and... monitor my own consumption of cigarettes and then wean myself down. Sometimes you don’t even realise how much you’ve smoked. If I had my phone telling me how many I’ve had, then I’m going to be like, ‘oh, okay,’ and that would help me keep track.

FG24, Mixed group participant

In addition to liking an app due to its tracking and accessibility, LGBTQ+ YYA would like to see a way to connect with others within their community who are also trying to quit smoking. This type of networking system would allow app users to encourage each other, share tips and tricks, and know there are others like them undergoing similar experiences. This could also be an avenue to connect with professionals and counselors, as this would be an easy way to access these professional resources.

If it were to heavily play into the community-base where it’s not just you on your phone and you get reminders, but if you were able to connect with other people, within a marginalised community, I think that would be great. I think (if) you could specifically reach out to certain groups, you know, trans people supporting trans, people of colour supporting people of colour... I think that would be a great option.

FG21, Mixed group participant

I do like getting access to counselling services because it’s so hard to get those resources, maybe there could even be a counsellor linked up in the app.

FG23, Mixed group participant

The next positive factor was tailoring of the app to the LGBTQ+ community, and this was considered very important to FG participants. In general, participants felt that an LGBTQ+-specific app would be superior to a general app, as the community would be friendly to those with LGBTQ+ orientation. There could be considerations embedded toward the struggles LGBTQ+ persons may have been through such as victimization. LGBTQ+ role models could also be prominently featured to influence smoke-free behavior.

Assuming that it’s customised to LGBTQ (and) it incorporates the kinds of struggles that we’ve lived through, it wouldn’t be any average quit-smoking app. The fact that it’s specific to a community... the fact that it’s LGBTQ-specific, that would help us more than if it was just a general quit-smoking app.

FG05, Mixed group participant

The fifth factor revolved around apps having the ability to link and share information via other social networks (eg, Facebook, Twitter). Participants felt that integration with such networks was a vital factor, as they would be able to share their experiences to garner additional support. It is important to note that participants felt that linking to a social network should be a choice, not a mandatory feature within the app.

Integrating it with the social media is definitely a great thing to do because they can always fall back to Facebook, Twitter, etc. And through this, people can get to share their experiences and keep an update and tell whatever experiences they may have to share. So it’s like ongoing support.

FG09, Lesbian group participant

Another essential feature that participants felt a quit smoking app should possess is the ability for the app to be personalized and customized by the user. For instance, participants shared that they could personalize the quit smoking goal in steps that were achievable to them. Further, the idea of uploading and posting personal pictures was revealed as a motivational tool that could be achieved through customization.

I think (the) key is that folks can personalize their goal and how they want to record and achieve it and see it monitored. Yes, that’s it.

FG21, Mixed group participant

Other reasons that were shared for liking a mobile app included that it would make individuals accountable to themselves, there would be no pressure to use it as it is more casual, it is preferable for those who wish for more privacy (as opposed to group counseling sessions), the intervention would appeal to the youth or young adult population, the idea itself is innovative and modern, and many individuals use other apps that are already successful.

#### Concerns Over the Use of a Mobile App for Smoking Cessation

Even though attitudes toward a culturally tailored quit smoking app were primarily positive, a number of concerns were raised by participants. The most important concern shared was that not all individuals possess a mobile phone, use social networking sites, or use apps.

I know everybody kind of has smart phones but some people don’t and I guess you kind of have to acknowledge that area of our community that doesn’t have access to these things. So it completely takes them out of the picture like if you’re completely [offline], you’re not even aware of it type of thing.

FG09, Lesbian group participant

Further, even though this idea was considered to be “awesome,” “exciting,” and “cool,” several participants felt that they might lose interest quickly. This was concerning, as the effectiveness of a quit smoking app on its own would be reduced if users do not access the resource.

It sounds like a good idea but I think it’s, kind of, like, fitness apps or something. They’re there, you know; you record the calories you eat for, like, the first few days you download it. And then after, it kind of loses the appeal. It’s like any app that you would download on your phone. So I think... it sounds like a good idea. But I think, for most people, it might just lose that interest after a while in having to go in there and, like, record stuff.

FG02, Gay group participant

The app market is currently filled with numerous quit smoking apps, some of which are at a significant cost for the user. Participants were concerned that a culturally tailored app would be in competition with other apps, unless properly marketed and shared through appropriate channels for the LGBTQ+ community.

The only thing with apps though, probably just being cynical again, but I feel like apps, it’s like such a saturated environment, there’s so many apps out there, I have so many apps on my phone I don’t use, like just pages on pages, like I will just scroll through, like I never use them. So I think it would be important that it had that kind of integrity behind it, and a good design behind it, to start out with, because like you were saying, there’s so many apps and there’s so many repeats of apps. So you would have to get a really good one out on the market first.

FG14, Lesbian group participant

Lastly, although the privacy offered by an app was a critical benefit, participants highlighted how this might make it easy to cheat and lie about progress to oneself.

You could be smoking and posting, oh, I’m not smoking – and you have the cigarette in your mouth still... And when it comes to lying to the app, like, you’re only really lying to yourself. That’s who you’re really lying to, you know? And also, I don’t know, I think it would be effective. I would use it.

FG02, Gay group participant

They don’t have to be sincere or genuine with the amount of cigarettes they smoked. The time they smoked. How often they smoked or even how often they didn’t smoke seeing that you can set up your goal... and then after that goal return back to your old habits. You’ve accomplished your three-month goal so three months is up now so back to the (old habits).

FG20, Trans group participant

#### Suggestions to Incorporate Into a Mobile App

Participants voiced a number of suggestions that would improve a quit smoking app intervention. Many of these suggestions stemmed from the concerns identified by participants and were offered as solutions to these concerns.

Several participants shared that some method of distraction should be included in the quit smoking app to help deal with cravings. This could be in the form of games or various mind puzzles that keep the smokers’ hands occupied.

If there was a bunch of games on the app that were there to distract you from smoking, (you could) go play five minutes of a quick game instead of smoking.

FG07, Mixed group participant

I think if there were some games on it, because that's one of the things—if my hands aren't busy then I'm going to smoke—so have something kind of fun like that.

FG16, Queer group participant

Another key addition proposed was that there should be a feature that provides rewards to the app user. Multiple reward ideas were proposed, such as virtual awards through the app, quit smoking contests using the app with associated prizes, and coupons or other incentives. Participants even commented that organizations who want smokers to quit may be willing to sponsor rewards through the quit smoking app.

If the quitting process takes a full year, unless you have some contests going on and perks associated with it, it (won’t) work. You can get a lot of people to sponsor prizes, like hospitals, doctors’ offices, and lotteries that are already trying to get people to quit smoking. There are a lot of organizations that are trying to get people to quit and I think they would support it with some prizes and stuff like that.

FG01, Gay group participant

Participants also thought it vital that any quit smoking app tailored to the LGBTQ+ community have a Web-based version or a version that operates even when there is no access to the Internet. A key concern that contributed to this idea was that many LGBTQ+ individuals are in poverty or homeless, and that there should not be an assumption that all potential users may have access to a mobile phone. It was suggested that the quit smoking app program also have print resources available (eg, pamphlets).

I feel like there would need to be a website equivalent with it (for) people who don’t have access to smartphones but do have access to public libraries. A lot of smokers are LGBTQ and a lot of LGBTQ are in poverty and homeless. The people that you want to access might not be able to access the program.

FG14, Lesbian group participant

Other improvements recommended that apps should incorporate a notification feature that reminds individuals to utilize the app and provides encouragement to the user.

It would be more effective if they send you those notifications every couple (of) hours. So then you have constant updates, and it’s a constant reminder. Every time my phone rings or dings I instantly pick it up. Once that would go off, I’d be like, ‘oh, okay, my lungs are starting to clear – that’s great.

FG02, Gay group participant

Further, personalization should allow users to create personal challenges that are relevant and achievable to them (eg, day 1 goal—go for a 20-minute walk around the block). Finally, there is need for the app to be positive, engaging, and cool, and it should be provided at no cost to the user.

Other considerations for the development and implementation of a tailored quit smoking app included a range of design factors that relate to the ease of use and quality of experience of using such an app. Participants indicated that the finished product should: be of high quality and bug free; protect individual app users’ privacy at all times; be available for all types of devices, such as Black Berry and not just Apple and Android devices; and lastly, be free of charge to use. Participants noted that ultimately, the effectiveness of such a tool was dependent on the app users’ level of dedication and desire to quit: “I don’t think any of these ... ideas, would help me quit, or even... (motivate me) to quit smoking.” [FG13, Bisexual group participant]

## Discussion

### Principal Findings

This study was the first to explore the perceptions of a large group of urban, Ontario LGBTQ+ YYA smokers and recent quitters about a culturally tailored smoking cessation app. The purpose was to discover what components of a mobile app smoking cessation program may resonate with LGBTQ+ YYA and what concerns and suggestions they may have about cessation apps. This addressed an important gap in the literature, as we sought input from a priority population with high smoking rates.

The overall findings revealed that LGBTQ+ YYA are quite enthusiastic about the role of mobile apps for smoking cessation. We learned that mobile apps could be harnessed for use within the LGBTQ+ community in a culturally tailored app that was specific and relatable, rather than being geared toward the general population. Other research has found that young persons are cautiously optimistic about the role of apps in health behavior change [19]; however, since that time, the explosion of apps in the market has exponentially increased and gained popularity. More recent research indicates that many current smokers would be interested in using a smoking cessation app [21].

FG participants offered reasons as to why a mobile smoking cessation app would work, including accessibility, portability, tracking and awarding progress, connecting with other community members and social networks, tailoring, notifications, and opportunity for customization. The reasons or preferences for the mobile phone app expressed by participants align with research on persuasive design where it has been shown that self-care technology such as a mobile phone app for smoking cessation should show understanding, persuade users to do the right thing, motivate behavior change, and provide rewards and appraisal for appropriate behavior as well as social support [36,37]. The feedback provided by a mobile phone app has the potential for informing, enabling, motivating, self-regulating, and guiding smokers in their efforts to successfully quit smoking [38].

The preferences for a mobile phone app were often cited in relation to experiences with other smoking cessation interventions that often require in-person meetings or appointments at significant time, travel, and cost to the individual. This finding adds to previous research that indicates that smokers are interested in finding help for quitting in new, more accessible mediums instead of support from quitlines, group cessation classes, or health professionals [39]. Of interest, LGBTQ+ YYA desired communication with other LGBTQ+ persons and community members, over that of health care providers, but through the app medium. However, some participants cited the importance of health professionals and counselors who should be linked with the app, as they are a key resource that can be difficult to access.

We also found that participants placed importance on privacy of their information, as individuals were concerned about protecting their LGBTQ+ identity, smoking status, and quit smoking progress. Although desire for “peer” social support was a highly expressed need from participants, “peer,” in this instance, referred to other anonymous LGBTQ+ YYA who are going through the same or similar quit smoking process, rather than their friends and family. Thus, private, as opposed to public, forums or chat spaces were what was desired among this specific group to help them feel safe to openly discuss their quit smoking progress.

Although some research indicates that smokers may not find games to be the most appealing feature [21], in this study, participants felt that gamification was an important form of distraction to the app user as a coping mechanism for cravings. This was critical in the minds of the participants and is supported by evidence demonstrating that assistance with cravings or provision of coping mechanisms such as distractions is central to successfully stopping cigarette addiction [40].

Lee et al (2014) have suggested that nontailored interventions may work as well as tailored for LGBTQ+ smokers [11]. However, FG participants indicated that a mobile phone app tailored specifically for LGBTQ+ was important. Specifically, respondents indicated that it was important that LGBTQ+ were involved in the creation of the app to ensure that it incorporated real-world experiences of the community. Moreover, there was an expressed desire to incorporate LGBTQ+ role models and the potential ability to connect with other LGBTQ+ YYA supports, such as LGBTQ+ counselors and other LGBTQ+ YYA peers (eg, trans youth supporting trans youth) who are going through the quit smoking process. Participants indicated that these specific LGBTQ+ tailored components would have a lot of influence and provide more incentive for use than a general quit smoking app. Considerable public health research has demonstrated the effectiveness of both targeted health promotion messages for population subgroups and individually tailored programs [41,42]. For example, personalized feedback can be adjusted to participants’ efficacy level and the unique impediments in their lives, and as a consequence, a smoking cessation app that can be tailored to LGBTQ+ smokers will encourage adoption and reach of the intervention as well as improved cessation outcomes. In addition, recent evidence based on the content analysis of mobile phone apps for smoking cessation suggests that tailoring of apps is positively related to user-rated quality [33].

Participants recognized the importance of social support in quitting smoking and commented on how the app could connect with others within their community. The personal connections that can be formed through a mobile phone app or social media can be viewed as social support, and social support is effective in helping people quit smoking [43]. Further, a community of LGBTQ+ app users may provide the opportunity for smokers to work together to quit smoking and improve the quality of their lives, as research on the effect of social media has revealed that sites, such as Facebook, can be harnessed for supporting young adults who are trying to quit smoking or have become smoke-free [44].

Finally, evidence in support of smoking cessation mobile phone apps is nascent. However, evidence is quickly building with a number of recent study protocols to test the effectiveness of mobile phone apps for smoking cessation as well as reviews of app content for evidence-based behavior change strategies [22-29,31-33].

### Limitations

This research had several limitations. The perceptions shared by FG participants were discussions of a hypothetical app use or experiences with previous app use situated in a particular time frame. It is unknown whether these speculations may change when an actual app is tested and used by the target population. Additionally, we did not probe participants on specific app features but allowed discussion to evolve organically so that participants could identify what was most important to them. However, participants in various FGs may have initiated conversation on features that others never spoke of. Third, we were unable to obtain the same number of participants within lesbian, gay, bisexual, transgender, and queer groups, and had to create mixed FG sessions. In addition, analysis for this paper was not conducted separately for each group, so it is unknown whether perceptions vary between LGBTQ+ groups. Further, more young adults than youth partook in this study. Finally, it is unknown whether the LGBTQ+ YYA in our sample will generalize to LGBTQ+ smokers in other communities and countries, as the context of our sample came from urban areas where services are typically available for those identifying as LGBTQ+. However, due to the paucity of research on YYA who identify as LGBTQ+, this research sheds light on the perceptions and opinions of this subpopulation group.

### Future Research

Although the consultation with LGBTQ+ young adult smokers shows promise [21-24], future research is needed to determine whether mHealth interventions can help individuals induce behavior change [45] such as quitting smoking. The LGBTQ+ YYA in this study were very interested in an app, so further research is needed to determine whether this population can benefit from a mobile-based smoking cessation intervention with social media connections. It would be useful to study actual app use and whether the findings that emerged from this study occur in a real-life situation. LGBTQ+ YYA should also be involved in the development of a mobile app through participatory research. Finally, additional research is needed to investigate the perceptions of various sexual and gender minorities to determine whether the differences between groups require tailoring specific to each subgroup. Furthermore, in addition to the need for assessing the effectiveness of apps in terms of abstinence from smoking, other important outcome measures such as increased quit attempts should be assessed, as Zhu et al (2012) have demonstrated that increased numbers of quit attempts are key to eventually quitting [46]. Once demonstrated as cost-effective and for whom, policy makers can determine how best to integrate mHealth and social media interventions within comprehensive tobacco cessation systems [31,33].

### Conclusions

mHealth interventions are increasingly popular for research and are a current, contemporary tool with potential to reach many individuals worldwide. Given the lack of consideration in the literature of LGBTQ+ YYA cessation interventions, this formative research contributes to knowledge in regards to what this population sees as important and beneficial in quitting smoking. We found that a mobile app intervention was viewed positively as a potential smoking cessation tool, and this highlights the need to develop such an app with LGBTQ+ YYA involved in the process. In addition to summative evaluation of a mobile phone cessation app’s effectiveness for LGBTQ+ YYA, additional formative research on the development and implementation of a mobile phone app is necessary to address knowledge gaps.

## Appendix

Multimedia Appendix 1 LGBTQ+ project: focus group script and questions.

Multimedia Appendix 2 Demographic and smoking characteristics of focus group participants.

## Abbreviations

FG: focus group
LGBTQ+: lesbian, gay, bisexual, trans, queer, and other sexual minorities
mHealth: mobile health
YYA: youth and young adults
