# Supplementary material for: Perceptions Toward a Smoking Cessation App Targeting LGBTQ+ Youth and Young Adults: A Qualitative Framework Analysis of Focus Groups
Source: JMIR Public Health Surveill. 2016 Nov 18;2(2):e165. doi: 10.2196/publichealth.6188 (PMC5135733; doi:10.2196/publichealth.6188)
Supplement: Multimedia Appendix 1 [file publichealth_v2i2e165_app1.pdf]

LGBTQ+ project: focus group script and questions

Welcome and Introductions

Welcome everyone and thank you for coming. My name is [name] and I will be your facilitator for this focus group session. We are working closely with some universities, Rainbow Health Ontario, and the Canadian Cancer Society to develop ways to help LGBTQ youth and young adults (lesbian youth; lesbian young adults; gay youth; gay young adults; bisexual youth; bisexual young adults; trans youth; trans young adults) quit smoking or better yet, to not start smoking at all. We have [name] here from Rainbow Health today. This is a really important area to address because LGBTQ youth and young adults are almost twice as likely to be smokers compared to non-trans and straight people of the same age and because smoking is extremely harmful to our health [if asked, 22% vs. 11% for youth as per the 2012 Atlantic Canada Drug Use Survey; 36% vs. 17% for young adults].

The purpose of this focus group is quite simple - we want your input and feedback. You know best what might work and what won't work. Our discussions will have two parts. First, we want to learn a little more about what you think about smoking. Knowing this will help us with the second part of this focus group at which point we would like to get your input on a scenario for encouraging quitting. I will be keeping us on track with the use of my [phone/watch] as a timer. This is to make sure we get through all the materials and we don't keep you for longer than we promised.

Before we begin, I just want to remind you of a few things. You may choose to not answer any question that you are asked. This discussion group is being audio-recorded to make sure everything is captured accurately; we also have a note-taker here. All information you provide is confidential although we may use some anonymous quotes when we share what we have learned. These are a few of the ground rules for this focus group: 1) please be respective of everyone's perspective; 2) one person speaking at a time, please; 3) we have 1.5 hours allotted for this focus group and I will be keeping us on schedule 4) there are no right or wrong answers; we are looking for your opinion and feedback; We also please ask that you don't share what other people say once this focus group is over. Your $50 will be handed out to you at the end of this session.

Does anyone have any questions before we begin? [Note-taker – please write down any questions that are asked including initials of who asked]

Ok – I would like to start this session by hearing from each of you. Can everyone please introduce themselves and share what movie they last watched. When you introduce yourself, please use the name that you wrote down when you registered today and say

your preferred gender pronoun (e.g., she/he/him/her). [1 minute per person max; 10 minutes max- facilitators, also please respond to this question so the group can warm up to you as well].

Intervention Ideas for Feedback

OK Great! Let's get started! Now, we're going to switch gears and ask for your input on an idea that may help LGBTQ youth and young adults quit and stay smoke-free.

I'm going to hand out a scenario. We're going to read it together and then we're going to discuss it. Does anyone have any questions?

While I'm reading the scenario, please write down any questions or thoughts you have and we can discuss them after I'm done reading. Again, there are no right or wrong answers. We simply want to get your feedback.

Scenario: Mobile Health Techniques

Do you own a smartphone? Ever play Candy Crush or use Instagram? What if there was an app designed specifically for LGBTQ youth and young adults that could help you quit smoking? For example, this quit smoking app would allow you to create an individualized quit plan where you can set a quit date, it would provide feedback on how you're doing, record what triggers you to smoke, and give you tips on how to remain smoke-free, as well as links to counselling services. One of the advantages of the app would be access to a peer support network which would connect you to other LGBTQ peers who are also trying to quit or who have already stopped smoking.

The app would be part of a bigger social media campaign that would include a webpage, Facebook page, Youtube videos, and Twitter feed with access to more detailed educational resources about smoking and quitting, for example, nicotine replacement therapy, like gum or the patch. LGBTQ role models would promote the campaign.

Questions:

1. How do you feel about a smartphone app and social media campaign for people your own age that are also LGBTQ, who smoke, and who want to quit smoking?
2. If a smoke-free app was customized to LGBTQ youth and young adults would you use it? Why or why not?
3. What are some things that you like about a smartphone app and social media program?
4. What are some things that you don't like about it?
5. Has anyone tried any other quit smoking apps? If yes, which ones? Did you find them useful?

Closing script

This concludes our time together. I would like to thank you for your time. Please leave the scenario descriptions on the table as we will be collecting them. On a piece of paper in front of you, can you please indicate how you found out about this focus group (poster, word of mouth, Facebook ad, Facebook page, SOY). Your thoughts and opinions are greatly appreciated.

Before you leave, please see [name], who will give you instructions on receiving your $50.
