# Supplementary material for: Perceptions Toward a Smoking Cessation App Targeting LGBTQ+ Youth and Young Adults: A Qualitative Framework Analysis of Focus Groups
Source: JMIR Public Health Surveill. 2016 Nov 18;2(2):e165. doi: 10.2196/publichealth.6188 (PMC5135733; doi:10.2196/publichealth.6188)
Supplement: Multimedia Appendix 2 [file publichealth_v2i2e165_app2.pdf]

Demographic and smoking characteristics of focus group participants (N=204).

CHARACTERISTICS	N (%)	CHARACTERISTICS	N (%)
**AGE (YEAR)**		**Have you smoked 100 cigarettes?**	
16-17	18 (8.8)	Yes	186 (91.2)
18-29	186 (91.2)	No	16 (7.8)
Total	204 (100.0)	Missing	2 (1.0)
Mean age	23	Total	204 (100.0)
**GENDER**		**Ethnicity**	
Female	85 (39.0)	Aboriginal	25 (10.4)
Male	58 (26.6)	Black/African/Caribbean	46 (19.1)
Trans female	8 (3.7)	Central Asian	1 (0.4)
Trans male	15 (6.9)	East/South East Asian	16 (6.6)
Two-spirit	9 (4.1)	Latin America	12 (5.0)
Queer	32 (14.7)	Middle Eastern	7 (2.9)
Intersex	1 (0.5)	South Asian	11 (4.6)
Other	10 (4.6)	White	115 (47.7)
Total[a]	218 (100.0)	Other	8 (3.3)
		Total[a]	241 (100.0)
**SEXUAL ORIENTATION**		**Education**	
Lesbian	27 (12.9)	Some high school (currently enrolled)	25 (12.3)
Gay	54 (25.8)	Some high school (not currently enrolled/not completed)	21 (10.3)
Bisexual	57 (27.3)	Completed high school with diploma	78 (38.2)
Queer	51 (24.4)	College degree[c]	35 (17.2)
Intersex	5 (2.4)	University degree[c]	40 (19.6)
Pansexual[b]	10 (4.8)	Graduate degree (Masters or PhD)	4 (2.0)
Other	5 (2.4)	Missing	1 (0.5)
Total[a]	209 (100.0)	Total	204 (100.0)
**HOW SOON AFTER WAKING DO YOU SMOKE?**		**Housing**	
<5 minutes	25 (12.3)	Living with parent	59 (25.2)
6–30 minutes	50 (24.5)	Rented or owned	118 (50.4)
31–60 minutes	31 (15.2)	Homeless	12 (5.1)
>60 minutes	64 (31.4)	Social housing	17 (7.3)
I don’t smoke	17 (8.3)	Couchsurfing	25 (10.7)
Missing	17 (8.3)	University/College residence	3 (1.3)
Total	204 (100.0)	Total[a]	234 (100.0)
**CURRENTLY SMOKE?**		**Years lived in Canada**	
Daily	113 (55.4)	0-1 years	12 (5.9)
Occasionally	58 (28.4)	2-5 years	17 (8.3)
Recent quitter	30 (14.7)	6-10 years	12 (5.9)
Missing	3 (1.5)	Over 10 years	163 (79.9)
Total	204 (100.0)	Total	204 (100.0)
**INTEND TO QUIT IN THE NEXT 30 DAYS**		**City**	
Yes	53 (26.0)	Toronto	156 (76.5)
No	32 (15.7)	Ottawa	43 (21.1)
Don’t know	89 (43.6)	Other	2 (1.0)
N/A	13 (6.4)	Missing	3 (1.5)
Missing	17 (8.3)	Total	204 (100.0)
Total	204 (100.0)		

[a]The total number reflects the number of responses; some participants selected more than one response.

[b]Including gray-A pansexual and pansexual demisexual.

[c]Those who said “some college” or “some university” were recoded into “college” or “university.”
